# The impact of bacterial exposure in early life on lung surfactant gene expression, function and respiratory rate in germ-free mice

**DOI:** 10.3389/frmbi.2023.1085508

**Published:** 2023-03-06

**Authors:** Kenneth Klingenberg Barfod, Julian Chun Lui, Signe Schmidt Kjølner Hansen, Sreyoshee Sengupta, Line Sidsel Fisker Zachariassen, Axel Kornerup Hansen, Jorid Birkelund Sørli

**Affiliations:** ^1^ Department of Food Science, Faculty of Sciences, University of Copenhagen, Frederiksberg, Denmark; ^2^ Department of Veterinary and Animal Sciences, Faculty of Health and Medical Sciences, University of Copenhagen, Frederiksberg, Denmark; ^3^ Section on Growth and Development, Eunice Kennedy Shriver National Institute of Child Health and Human Development, National Institutes of Health, Bethesda, MD, United States; ^4^ Biotech Research & Innovation Centre, Copenhagen, Denmark; ^5^ The National Research Center for Work Environment, Copenhagen, Denmark

**Keywords:** germ-free, lung gene transcription, plethysmography, lung surfactant, microbiome

## Abstract

Early-life changes to lung and gut microbiota have been linked to alterations in immune responses that may lead to pulmonary diseases later in life. Associations between early-life microbiota, germ-free status, lung gene expression, lung development and function are not well described. In this study, we compare early-life lung gene transcription under germ-free and different perinatal microbial exposures, and analyze with a predetermined focus on lung capacity and lung surfactant. We also analyze the later-in-life physiological measures of breathing patterns and lung surfactant function between the germ-free, gnotophoric and gnotobiotic offspring. To achieve this, we kept pregnant BALB/c germ-free mice in separate germ-free isolators until exposure to either A: no exposure (GF), B: *Bifidobacterium animalis ssp. Lactis* (BI04) or C: full cecum content harvested from other female SPF mice (Cecum). Subsequently, perinatally exposed offspring were used for the analyses. Lung tissue transcriptomics analysis was done at postnatal day 10 (PNday10) at the first phase of lung alveolar development. Head-out plethysmography for breathing pattern analysis was performed on the siblings at PNday23 followed by lung surfactant collection. The function of the collected lung surfactant was then analyzed *ex vivo* using the constrained drop surfactometer. Our results show that lung transcriptomics had differentially expressed genes related to surfactant turnover between groups and sex at PNday10. They also show that the GF and BI04 animals had lower respiratory rate than Cecum mice, or compared to age-matched specific pathogen-free (SPF) reference animals. We also see changes in lung surfactant function *ex vivo.* The overall conclusions are that 10-day-old GF mice do not have a markedly different lung gene transcription compared to gnotophoric or gnotobiotic mice, but genes related to surfactant metabolism are among the few differentially expressed genes. We show here for the first time that early-life microbiome status correlates with early-life surfactant-gene transcription and to later-in-life lung surfactant function and associated respiratory-rate changes in mice.

## Introduction

Reduced lung capacity and function early in life has potential consequences for respiratory health later in life (Phelan et al., 2002; Stern et al., 2008; Hancock et al., 2010). Perinatal microbiome alterations have been investigated in relation to the development of chronic and developmental lung diseases such as asthma and bronchopulmonary dysplasia (BPD) (Tirone et al., 2019; Ashley et al., 2020; Casado and Morty, 2020; Cereta et al., 2021). Most studies focus on the microbiome impact on the developing immune system and resistance to pathogens (Herbst et al., 2011; Willers and Viemann, 2021). Only few studies have investigated perinatal microbiome influence on lung development, physiology, function and structure (Yun et al., 2014; Decrue et al., 2020; Schlosser-Brandenburg et al., 2021). Most studies are largely descriptive in nature, and even though there is increasing evidence that the human microbiome is involved in the development and progression of chronic respiratory diseases, more mechanistic studies and insights are warranted (Gosens et al., 2020).

Mouse studies have mostly used immune-competent mice treated with antibiotics (Russell et al., 2015) or they compare germ-free (GF) to specific pathogen-free (SPF) mice (Remot et al., 2017; Dolma et al., 2020). GF mice are thought to develop differently than SPF mice. In mice from a GF background that have been without bacterial stimuli throughout their entire development, organs, gastrointestinal function and immune system are thought to develop differently (Thompson and Trexler, 1971; Sommer and Bäckhed, 2013). These developmental differences between GF and SPF mice have been poorly described; especially for the respiratory system. Furthermore, the question of whether early-life microbiome status can affect later lung surfactant function has not yet been investigated (Bernhard, 2016). Lung surfactant consists of a complex mixture of phospholipids and proteins that lines and stabilizes the alveolar respiratory surface. The main function of lung surfactant is to reduce the surface tension at the air-liquid interface, and poor lung surfactant function can affect respiratory rate due to higher surface tension. It also acts as a first line defense against respiratory pathogens (Clements, 1957; Bernhard, 2016; Olmeda et al., 2017).

In this study, we compare offspring that have been exposed perinatally to different microbial stimuli during pregnancy and through weaning with other fully GF offspring. The offspring were all born from originally GF dams in order to compare them better. We study early-life lung gene transcription, breathing patterns and lung surfactant function in adolescent GF mice, compared to mice that were perinatally exposed to either a single strain of *Bifidobacterium* (BI04) or exposed to a full cecum microbial community by fecal matter transplant from donor adult SPF dams (Cecum). The BI04 strain is a well-documented probiotic with beneficial early-life effects. The strain is currently being tested for use in asthma and allergy treatments, and to reduce respiratory tract infection risk. The BI04 strain was also used in our previous microbiome studies (Ouwehand et al., 2009; Weiss et al., 2010; Barfod et al., 2015; Hui et al., 2021).

Our hypotheses were that perinatal exposure to bacteria would influence lung gene expression early in life in regards to mucus production, lung capacity, physical development and breathing patterns later in life. We also hypothesize a possible specific probiotic influence from the BI04 strain. Furthermore, special focus was on the hypothesis that mice born from GF mice (GF animals) compared to mice born from GF dams exposed to full microbiome (Cecum animals) would influence lung surfactant function, thus indicating for the first time that lung surfactant function is correlated to the microbiome.

## Methods

### Aim

Our aim was to compare possible early-life lung transcriptomic differences in genes related to lung development, lung surfactant and capacity induced by microbial stimuli to GF mice.We also wanted to explore the possibility that the early-life microbiome status could influence surfactant properties (*ex vivo*) and respiratory parameters such as rate (*in vivo*) later-in-life.

### Design and setting of the study

We compared three treatment groups of mice: 1) GF mice born from untreated germ-free dams (1=GF), compared with offspring born from GF dams that during late pregnancy and weaning were exposed to either 2) an inoculum with single probiotic bacteria strain (2=BI04) or 3) a fecal matter transplant with full cecum material from non-GF SPF dam donors (3=Cecum). We compared transcriptomic data from lung tissues from a subset of offspring (M+F) when 10 days old using two different bioinformatics tools for pathway analysis. Additionally, the transcriptomic data were analyzed and compared with two pre-made “short lists”. These were lists of interesting genes associated with total lung capacity and surfactant-relevant genes from two previous studies published by others (George et al., 2017; Olmeda et al., 2017). When the remaining sibling mice were 23 days old, we recorded their breathing patterns and respiratory rate and collected their lung surfactant for functionality testing. Surfactant from the GF, BI04 and Cecum groups was tested *ex vivo* with a constrained drop surfactometer (CDS) and referenced to breathing patterns and surfactant properties from a fourth group of normal age-matched SPF mice (SPF).

### Ethics

The experiments were carried out in accordance with the Danish Animal Experimentation Act (LBK no. 474 of 15/05/2014 and BEK no. 12 of 07/01/2016) and EU Directive 2010/63/EU on the protection of animals used for scientific purposes. The study was approved by the Animal Experimentation Inspectorate under the Ministry of Food, Agriculture and Fisheries of Denmark (License no. 2018-15-0201-01531), and health monitoring was performed according to FELASA guidelines (Mähler Convenor et al., 2014).

### Mice and husbandry

GF BALB/cAnNTac mice (Taconic, Germantown, NY) were housed in our AAALAC accredited germ-free (GF) facility (University of Copenhagen, Frederiksberg, Denmark) in HEPA-ventilated isolators (PFI systems, Milton Keynes, UK; pressure 110 pascal, 23°C) with free access to an irradiated Altromin 1324 diet (Brogaarden, Lynge, Denmark) and sterile water under a 12h light/dark cycle. The cages were provided with standard bedding, house and nesting material and were environmentally enriched with a wood stick for gnawing. GF status was tested both by culturing and PCR methods (Zachariassen et al., 2019). Microbiome manipulations were confirmed and all cecum content was visually inspected at necropsy (Grover and Kashyap, 2014). After time-mating with GF males, visibly pregnant females were transferred under sterile conditions to two separate empty sterile isolators (5-6 females/isolator) approximately at embryonic day 18 (E18), corresponding to the beginning of the 3rd trimester in humans (Blum et al., 2017). Adult females were inoculated orally with 100µl bacterial suspension and 900 µl were smeared on the abdomen and mammae a total of 5 times: Day E18, PNday0-1, PNday5, PNday10 and PNday15. Pups were indirectly exposed to the bacteria by the mammae smear and local environment. Specific pathogen free (SPF) background BALB/cAnNTac mice (n=10) (Taconic, Denmark) PN21-23 day old, were used as a reference group for head-out plethysmography and surfactant function only.

### Bacterial inoculums

*Gnoto-phoric (BI04) exposure:* 100 mg freeze-dried *Bifidobacterium animalis ssp. lactis BI-04* (ATCC SD5219) approximately 5e10 CFU from DuPont Nutrition & Health, Finland were mixed with 1:1 with sterile 80% glycerol to 1 ml in aliquots and kept at -80°C. *Gnoto-biotic(Cecum) exposure:* Ten SPF 20-week-old BALB/cAnNTac dams (Taconic, Denmark) were killed after weaning and full cecum content was collected and mixed into one inoculum 1:1 with sterile 80% glycerol, aliquoted to 1ml and kept at -80°C. The GF dams did not receive mock treatment with 80% glycerol.

### Lung tissue sampling and RNA extraction

PNday10-old mice were killed (n=10 per exposure group) and the lungs excised. A 3mm×3mm tip of the left lung was snap frozen in liquid nitrogen and kept at -80°C. RNA extraction on the lung tissue was carried out utilizing magnetic beads technology on a chemagic Prepito^®^ (Perkin Elmer, Waltham, Massachussets), as recommended by the manufacturers. Concentration and purity were measured with a NanoDrop1000 spectrophotometer, with all samples showing an A260/280 ratio between 1.9 and 2.1. RNA integrity was analyzed using a 2100 Bioanalyzer (Agilent Technologies) with Agilent RNA 6000 Pico Kit (Agilent Technologies), as recommended by the manufacturer. All samples used for RNAseq displayed RNA integrity number (RIN) above 7.

### RNA-seq library construction and sequence mapping

Sequencing libraries were prepared using a TruSeq Stranded mRNA Prep Kit (Illumina), without the polyA selection step. Sequencing was performed *via* a paired end 75 cycle on Illumina HiSeq 2500 (Molecular Genomics Core, NICHD). The RNA-Seq data have been submitted to the NCBI (https://www.ncbi.nlm.nih.gov/geo), with GEO accession number GSE201075. RNA-Seq reads were trimmed with cutadapt (-AAGATCGGAAGAGCACACGTCTGAACTCCAGTCA -AAGATCGGAAGAGCGTCGTGTAGGGAAAGAGTGT -overlap 6 -q 20 -minimum-length 25) and aligned using STAR (2 pass alignment) to mouse mm10 reference genome sequences.

### Bioinformatics

Heat maps were then constructed using JMP 7 software (SAS Institute Inc., Cary, NC). The Reactome pathway knowledgebase (Gillespie et al., 2021) and Ingenuity Pathways Analysis Software 7.0 (Ingenuity Systems Inc., Redwood City, CA) was used to identify functional pathways. The analysis included genes that showed either an increase or decrease in transcription with adjusted p-value (corrected for multiple comparison) P<0.1 in DESeq2.

### Head-out plethysmography

PNday23 mice from the 3 exposure groups were placed in an ordered random fashion across runs, in whole body plethysmographs with head-out only, using the NOTOCORD-hem data acquisition software (Notocord Systems SA) to collect respiratory parameters; respiratory rate (RR), tidal volume (VT), time of inhalation (TI) and expiration (TE) and time-of-pause (TP) as described previously (Larsen et al., 2004). The fourth SPF reference group (n=10) were recorded on a separate run. The acclimatization period for all runs was 10 minutes followed by 30-minute breathing data capture.

### Lung surfactant collection and analysis

After breathing pattern analysis, the mice were killed and bronchoalveolar lavage (BAL) was performed as before (Barfod et al., 2013). After removal of cells, the lung lavage fluid was centrifuged to collect the large aggregates of lung surfactant at 20,000 x g, for 1 h at 4°C (Haczku et al., 2001). The lung surfactant was pooled by dam (n=1-4 per group, both sexes) to have enough sample to repeat measurements of each sample. The pooled samples were analyzed for phospholipid content using a phospholipids kit (kindly donated by Spinreact, Spain), and the phospholipid content was adjusted to 1.8-2 mg/ml. For each surface tension measurement, 10 µL was analyzed and the analysis was repeated until the sample was empty (1-8 times per pool). The number of measurements per group was GF=14 mice, BI04 = 21, Cecum=17, reference SPF mice=10. The lung surfactant was left at 37°C for 5 min prior to testing. Testing was done using a constrained drop surfactometer (CDS, BioSurface Instruments, United States) in which a droplet of lung surfactant is deposited on a sharp-edged pedestal, so that a surfactant film was formed at its air–water surface. The adsorbed lung surfactant film was pulse-cycled continuously to mimic breathing and surface tension values were obtained by analysis of the drop shape using the axisymmetric drop shape analysis software (ADSA) (Valle et al., 2015; Sørli et al., 2016). Several properties of lung surfactant were important for lung surfactant function during the breathing cycle; 1) the phospholipids have to rapidly cover the air-liquid interface (adsorption) when the surface area expands during inhalation, and 2) when the surface area is reduced during exhalation, the phospholipids need to efficiently pack to reduce surface tension (Autilio and Pérez-Gil, 2019). To analyze the adsorption surface tension, 10 µl of lung surfactant was placed on the pedestal and left for 60 sec while taking pictures. The average surface tension over the 60 seconds was reported as adsorption surface tension. To test how well the phospholipids pack and redistribute in the interphase during compression and expansion cycles, the drop was then increased in size by adding approximately 4 µL buffer, and then cycled at approximately 30% reduction in surface area at 3-second cycles. The number of cycles needed for the minimum surface tension to reach <5mN/m was counted. The compression was kept as similar as possible throughout the experiments to allow for comparison between groups.

## Statistics

### RNA-Seq data and statistical analysis

Differentially expressed genes between GF and Cecum or BI04 were identified with DESeq2. Because principal component analysis showed that sex (determined by RNA-Seq, looking for presence of reads in the Y chromosome) was by far the most significant confounding variable, we performed our analysis in two ways. First, we performed an overall analysis determining the effect of treatment (GF vs Cecum and GF vs BI04), with sex being a confounding variable. Second, we performed another analysis in which the male and female samples were analyzed separately for the effect of treatment. Genes were considered statistically significant with a fold change greater than 1.5 (or 0.585 after log2 transformation) and false discovery rate (FDR) <0.05. FDR was based on p-value adjusted for multiple comparison using the Benjamini Hochberg correction. Plethysmography: Two-way ANOVA and Tukey multiple comparisons of means as an average of 30 minutes of data capture with sex as a variable. Surfactant function: The data was analyzed using R-Studio Version 1.3.1056 and the package dplyr. According to the Shapiro-Wilks test, the data were not normally distributed, except for data for absorbance. Therefore, the Kruskal-Wallis test was used to test for a difference between groups for number of cycles needed to reach a minimum less than 5mN/m and for compression. There was a difference for number of cycles, thus we performed a pairwise Wilcoxon rank sum test to find different groups. An ANOVA was performed on the data for absorbance, and this showed a difference between groups, this was followed by the Tukey test to find differences between the groups.

## Results

### Non-sex differentiated transcriptomics results

A few genes were upregulated in GF compared to Cecum (17 genes), and BI04 (30 genes). A total of 8 genes were significantly upregulated in both comparisons: AU040320, Cox6b2 (Cytochrome C Oxidase Subunit 6B2), Fmn1 (Formin 1), Gm11399, Gm7638, Hist4h4 (Histone H4), Mid1 (Midline 1), and Sfi1 (SFI1 Centrin Binding Protein) (Chi-square P<0.001). Other genes were down-regulated in GF compared to Cecum (26 genes) and BI04 (24 genes). A total of 14 genes were significant in both comparisons: Ap1m2 (Adaptor Related Protein Complex 1 Subunit Mu 2), Arhgap19 (Rho GTPase Activating Protein 19), Arl2bp (ADP Ribosylation Factor Like GTPase 2 Binding Protein), C530043K16Rik, Cyp26b1 (Cytochrome P450 Family 26 Subfamily B Member 1), Fbxl3 (F-Box And Leucine Rich Repeat Protein 3), Gm33195, Gm37954, Hmbs (Hydroxymethylbilane Synthase), Mfap1a (Microfibrillar-associated protein 1A), Mfap1b (Microfibrillar-associated protein 1B), Nlrp5-ps (NLR family, pyrin domain containing 5), Prg2 (Proteoglycan 2, Pro Eosinophil Major Basic Protein), Shisa7 (Shisa Family Member 7), Stox2 (Storkhead Box 2), Zfp979 (zinc finger protein 979) (Chi-square P<0.001) (For all, see **Supplementary Table 1**.) (For volcano plots see **Supplementary Table 1A** GFvs BI04 and 1B GF vs Cecum.)

### Cluster analysis

**Figure 1:**
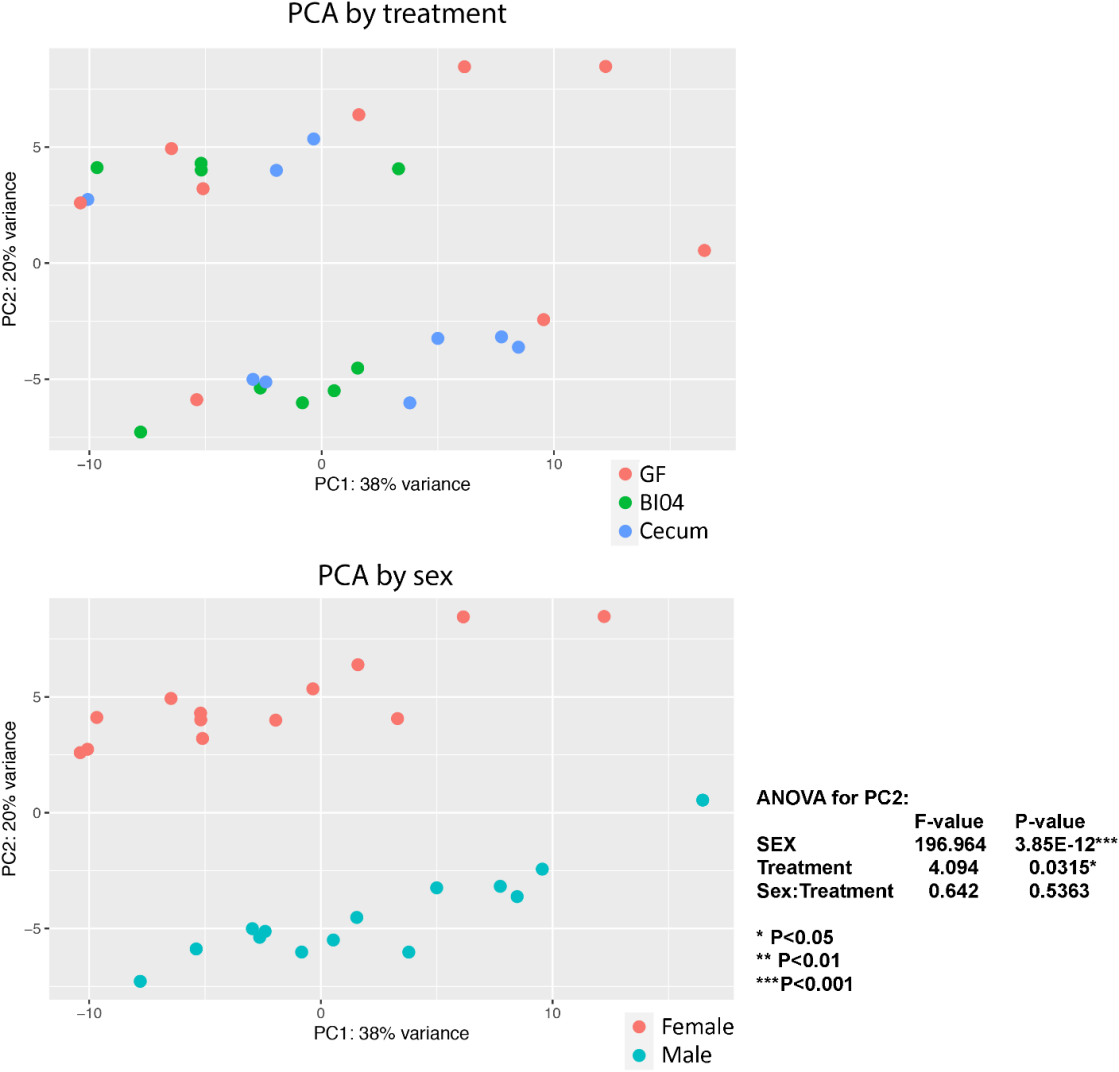
Shows lung tissue transcriptomics at PNday10 as PCA plots by treatment and sex. By Treatment: The samples clusters by perinatal treatment GF=Germ-free offspring, BI04=Offspring perinatally monocuture exposed to Bifidobacterium (BI04), and Cecum= Offspring exposed to full cecum community (p=0.0315), By Sex: The strongest clustering was according to sex, RED= Females and Blue= Males (p=0.0001).

PCA plots were completed by treatment and sex of the animals (**Figure 1**). Unexpectedly, at PNDay10 the major difference in lung transcriptomics was due to the sex of the animals, although the treatment effects were still significant. The samples clustered by treatment (p=0.0315), but sex of the offspring was the strongest difference (p=0.0001), without interaction between sex and treatment. Therefore, we performed all subsequent analyses separated by sex, where possible. (For separate sex differentiated PCA plots, see **Supplementary Figure 2**.)

### Sex-differentiated transcriptomics results

The common list of a total of 586 transcripts was significantly differentially expressed in at least one of two comparisons, i.e. when comparing GF to BI04 and Cecum animals. Of those, only 14 transcripts overlaped across all sex differentiated comparisons: Arl2bp, Sfi1, Mfap1a, Zfp979, Arhgap19, Gm37954, Fbxl3 (F-Box And Leucine Rich Repeat Protein 3), AU040320, Stox2, Mfap1b, Apbb2 (Amyloid Beta Precursor Protein Binding Family B Member 2), Gm33195, C530043K16Rik, and Hist4h4. Only eight transcripts were up or downregulated in Bi04 compared to Cecum: Apoa2 (Apolipoprotein A2), Gm10801, Gm10717, Gm10800, Ahnak (AHNAK Nucleoprotein), Tnk2os (Tyrosine kinase, non-receptor 2), Egfr (Epidermal Growth Factor Receptor) and Fbxl12os (F-box and leucine-rich repeat protein 12) (Chi-square P<0.001). (See Heatmap in **Supplementary Figure 3** at the end of the manuscript) and list with p-values in **Supplementary Table 2**.)

### Bio informatics analysis

We analyzed the transcription data in two different ways, with two different tools. The first approach was using the Reactome pathway knowledgebase, which is an open source, curated database of pathways and reactions in human biology (Gillespie et al., 2021). Using the sex-differentiated but compiled data for a Gene Ontology analysis, we found several lung surfactant-relevant functional pathways emerged. When comparing the significant transcripts between Cecum and GF mice the PANTHER, overrepresentation analysis showed the following, selected pathways: Defective CSF2RA causes pulmonary surfactant metabolism dysfunction 4 (SMDP4) (R-HSA-5688890), Diseases associated with surfactant metabolism (R-HSA-5687613), Surfactant metabolism (R-HSA-5683826) and Gene Ontology Biological Process Complete protein-lipid complex subunit organization (GO:0071825). (For complete table see **Supplementary Table 3A**.) When comparing the significant transcripts between BI04 vs GF, the results were less profound and consisted mostly of household gene pathways involved in cell proliferation and division. (For complete table see **Supplementary Table 3B**.)

The second bioinformatic approach used the Ingenuity Pathway Analysis Tool. The pathways were generated using QIAGEN Ingenuity Target Explorer (QIAGEN, Inc. https://targetexplorer.ingenuity.com/). We found 74 pathways, of which a few selected ones could be related to lung development and to the presence of bacteria when comparing Cecum animals with GF animals such as; “Regulation of eIF4 and p70S6K Signaling”, “Production of Nitric Oxide and Reactive Oxygen Species in Macrophages”, and “IL-12 Signaling” and “Production in Macrophages”. (For complete table see **Supplementary Table 4A**.) Again, when comparing the significant transcripts between GF and BI04, the results were less profound. We found 31 pathways consisted mostly of household gene pathways involved in cell proliferation and division. A few selected, possibly interesting pathways, stand out: “Antigen Presentation Pathway”, “IL-4 Signaling”, and “VEGF Signaling” as important for lung and immune development. (For complete table see **Supplementary Table 4B**.)

### A few genes matched our pre-made list of possible genes for lung capacity

Based on the publication by Leeme et al (George et al., 2017), we compared transcripts possibly related to lung capacity. They had found 34 genes consistently upregulated or down-regulated at day E18, P28 and P70 (**Supplementary Table 5**). The lung-capacity-relevant transcripts werebased on two different mouse strains: the C3H/Hej mouse, which grows to have about twice the lung capacity of the second strain, the age-matched JF1/MsJ mice. Inclusion on the list was increased lung transcripts in JF1/MsJ (JF1) (small lung) compared to C3H/HeJ (larger lung) mice at E18 (cut off for fold change ≥2 fold; false discovery rate <10%; total number of transcripts=45). In our transcripts, we found 10 out of 34 genes differentially expressed overlapping the prepared list of lung-capacity-relevant genes: Glrx3 (Glutaredoxin 3), Gm14403, 2610507I01Rik, Glp1r (Glucagon Like Peptide 1 Receptor), Scn3a (Sodium Voltage-Gated Channel Alpha Subunit 3), Nme7 ((NME/NM23 Family Member 7), A330076H08Rik, Alad (Aminolevulinate Dehydratase), Gpr137b (G Protein-Coupled Receptor 137B) and Hc (Hemolytic complement).

### Several lung surfactant relevant genes match our pre-made list

Based on the publication by Olmeta 2017 (Olmeda et al., 2017), we compiled a list of lung surfactant-metabolism-relevant genes (**Supplementary Table 6**). We found 17 out of the 23 lung surfactant relevant genes on this list were differentially expressed in at least one comparison of our data: SFTPA1 (Surfactant Protein A1), SFTPB (Surfactant Protein B), SFTPC (Surfactant Protein C), SFTPD (Surfactant Protein D), ABCG1 (ATP Binding Cassette Subfamily G Member 1), ABCA1 (ATP Binding Cassette Subfamily A Member 1), Bach2 (BTB Domain And CNC Homolog 2), CHKA (Choline Kinase Alpha), PLPP2 (Phospholipid Phosphatase 2), ABCA3 (ATP Binding Cassette Subfamily A Member 3), Csf2rb (Colony Stimulating Factor 2 Receptor Subunit Beta), Csf1r-ps (Colony stimulating factor 1 receptor), Csf2rb2 (Colony stimulating factor 2 receptor, beta 2), Pla2g2a (Phospholipase A2 Group IIA), Pla2r1 (Phospholipase A2 Receptor 1), Pla2g6 (Phospholipase A2 Group VI), Pla2g12a (Phospholipase A2 group XIIA).

### Respiratory rates are lower in GF and BI04 adolescent animals

We found that respiratory rates (RR) were lower in GF and BI04 animals compared to the similar RR frequency of Cecum and the SPF reference animals and what have been shown by others (Bernhard et al., 2001). The two-way ANOVA for RR were significant for treatment (p= 0.00671) but showed no statistical differences based on the sex of the animals or had any sex/treatment interaction. The average numbers of breaths (RR) were: GF(n=16)=230/min, BI04(n=19)=220/min, Cecum(n=19)=267/min and the SFP references group(n=10)=261/min. The Cecum animals had a statistically higher RR than GF and BI04 ((GF vs Cecum p=0.0550782, Cecum vs BI04 p=0.0069387)). There were no statistically significant differences between GF, BI04 or Cecum mice for any of the other recorded breathing parameters related to tidal volume (VT), time of inhalation (TI) and expiration (TE) and time-of-pause (TP) (Results not shown). The body weights were a little higher in the SFP age-matched references group with average body weight 16.1 g, whereas all mice originating from GF-background groups all weighed less with a comparable average GF=9.9g, BI04 = 8.9g, Cecum=9.5g, which was not significantly different statistically.

### Lung surfactant function differed between GF and BI04 animals

**Figure 2:**
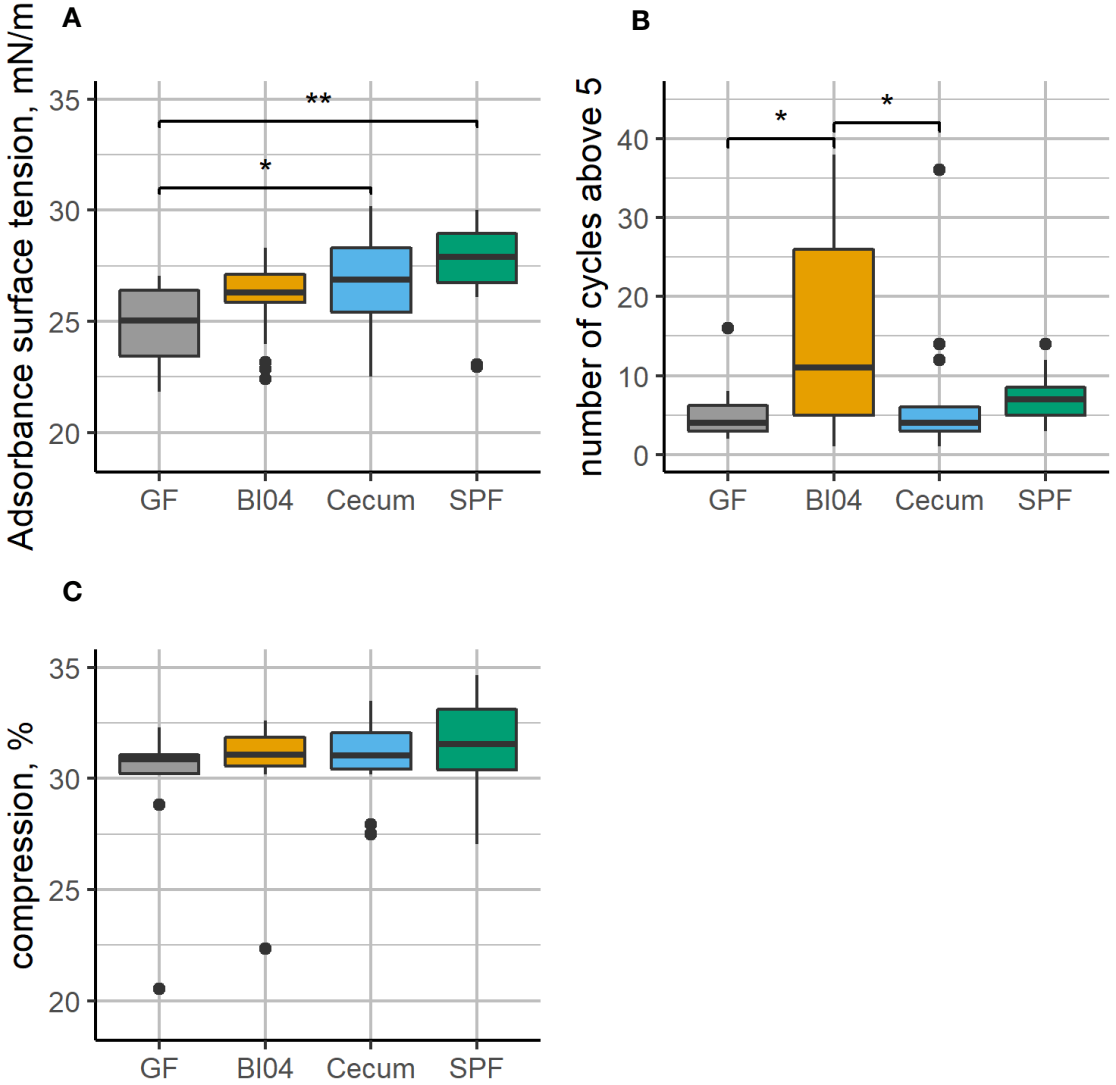
Lung surfactant from GF, BI04, Cecum exposed mice, and age-matched SPF mice was collected and analyzed using the constrained drop surfactometer. **(A)**: shows adsorption surface tension during the first 60 seconds, **(B)**: number of cycles before reaching minimum surface tension below 5 and **(C)**: compression in percent. The adsorption surface tension is similar, however, while Cecum and SPF have a higher adsorption surface tension than GF mice. The surfactant collected from BI04 exposed mice needed to be compressed more times before it reached a minimum surface tension less than 5mN/m compared to GF and cecum exposed mice. The compression was the similar for all groups (no statistical significant differences), which allows comparison between groups. *p<0.05, **p<0.01.

The adsorption surface tension of the lung surfactants collected at PNDay23 was affected (see **Figure 2A**) and shows an increasing pattern according to perinatal exposure, with GF mice having statistically lower adsorption surface tension than both the Cecum and SPF groups. Furthermore, the surfactant collected from BI04 exposed mice had a less well functioning surfactant (see **Figure 2B**) and had to be compressed more times before it reached a minimum surface tension of less than 5 mN/m compared to GF and Cecum exposed mice. The measured compression % of all lung surfactants was similar for all groups, with no statistically significant differences, which allows comparison of the surfactant function between groups (see **Figure 2C**).

## Discussion

GF animals have largely been used to study immune maturation in the absence of microorganisms and how this absence affects the morphological development of organs, however the effects on the lungs have been largely overlooked (Sommer and Bäckhed, 2013). We wanted to explore whether early-life microbial exposure could influence lung development and function. We hypothesized that lung gene expression of GF mice would be different to that of perinatally exposed offspring from a GF background. To this end, we exposed mice perinatally to a full ecologically complex microbiota and analyzed the transcriptomics changes in genes related to mucus production, alveolar development, lung surfactant metabolism and lung capacity. To elude any probiotic influences, we compared single strain exposure to that of GF and Cecum exposed animals. Furthermore, we wanted to investigate the early life microbial differences in relation to later-in-life breathing patterns and surfactant function.

The development of the lungs is not complete at birth, and at PNday10 the mouse lung is in the first phase of alveolarization (Mund et al., 2008). This early-life alveolarization is especially interesting in relation to BPD diseases research, which has been correlated to early-life lung microbiome in mice (Yang and Dong, 2020). Previously, it has been shown that GF mice have altered alveolar architecture compared to SPF and non-SPF mice (Yun et al., 2014). These observations indicated that microbiota could influence lung morphology and that higher bacterial abundance correlated with more and smaller alveoli. This was corroborated by transplanting murine-derived single-strain exposure of *Lactobacillus* spp. into the lungs of GF mice. These mice showed enhanced mucus production and significant differences in alveolar diameter and numbers (Yun et al., 2014). This has not been supported by others, and there are some conflicting publications that have shown no major physiological changes in lungs of GF mice (Remot et al., 2017).

Although it has previously been shown that there were no sex-differences in lung volume based on stereology at PNDay10 (Pozarska et al., 2017), we showed that sex had the biggest impact on transcription at PNday10 and we decided to analyze the data based on the sex of the animals, even though this gave us smaller group sizes. Our data do not show large lung transcription differences in GF animals related to lung architecture, immunity or mucus production at PNday10. The transcription of the gene encoding the main mucin in the lung (Muc5ac) has been shown by Remot et al. to be higher in SPF mice than in their GF counterparts (Remot et al., 2017). Yun et al. reported a reverse correlation of mucus production in the lungs with low bacterial abundance (Yun et al., 2014). Our data do not support differences in Muc5ac transcription between GF, BI04 or Cecum animals from GF-background at PNDay10.

The specific probiotic effects are small but largely relevant. We found 8 genes, mostly in female mice, that were upregulated or down regulated in BI04 animals compared to GF and Cecum animals: Gm10717 have been correlated to susceptibility to influenza A virus infection in mice (Krementsov et al., 2017). Gm10800 is related to defects in airway mucosal defense and pathogenesis of human chronic obstructive pulmonary diseases (Saini et al., 2014). Gm10801 has been shown to be relevant in intestinal epithelium maturation after antibiotic treatment in mice (Garcia et al., 2021). The apoa2 gene encodes a possible lung surfactant-relevant apolipoprotein and is part of the developing human lung transcriptome (Kho et al., 2010). A gene polymorphism in apoa2 has been linked to respiratory rhythmogenesis in mice (Gillombardo et al., 2017). The developmental emergence of the respiratory rhythmogenic neural circuits occurs during the last third of pregnancy embryonic day (E15) in mice (Chevalier et al., 2016).

The bioinformatics analysis of gene pathways and reactions compared to human biology does not reveal major shifts in the GF lungs compared to the groups with perinatal bacterial exposure. However, the few emerging differences between GF and Cecum-exposed mice were lung surfactant, dysfunction, metabolism and protein-lipid interaction relevant. The Ingenuity Pathway Analysis Tool also revealed a few differences between GF and the BI04/Cecum groups that are relevant to macrophage responses in the presence of bacteria and some that are important for lung and immune development, including IL-4 signaling pathway. Others have shown that the diversity lung microbiome are strongly correlated to “immune-tone” with baseline concentrations of inflammatory cytokines IL-1α and IL-4 (Dickson et al., 2018). Our pathway analysis shows that IL4 signaling is influenced by perinatal bacterial exposure and that il1α is less transcribed in male GF animals compared to BI04 and Cecum animals.

When comparing the list of genes for lung capacity derived from Leema et al. only 10 transcripts matched with the relevant genes for lung capacity: Glrx3, Gm14403, 2610507I01Rik, Gpr137b, Glp1r, Scn3a, Nme7, A330076H08Rik, Alad and Hc. The dysregulation of Glrx3 is associated with Idiopathic pulmonary fibrosis (IPF) asthma and COPD (Chia et al., 2020), and Gm14403 (predicted gene). The 2610507I01Rik transcript has been associated with ventilator−induced lung injury in mice (Xu et al., 2018). The Gpr137b is a possible regulator of in IL4 mediated M2 macrophage polarization in response to microbial recognition patterns (Islam et al., 2019). The Glp1r encodes the Glucagon-like peptide-1 receptor (GLP-1R) and it plays an essential role in normal lung function. In a rat model of pulmonary fibrosis induced by bleomycin, administration of liraglutide a GLP-1R agonist, decreased mRNA expression of collagen, hydroxyproline and other key enzymes. When restored, the ACE2 mRNA levels modulating the activities of the RAS components increased the production of surfactant proteins (SFTPa1, SFTPb, SFTPc) (Fandiño et al., 2020). We exposed perinatally, starting at embryonic day 18 (E18) corresponding to the beginning of the third trimester in humans. It is likely that we would have seen larger effects on transcription of lung-capacity-relevant genes if we had started bacterial exposure earlier in the pregnancy, i.e. from fertilization and implantation through placental development, as vascularization and embryonic organogenesis mostly takes place during the first trimester: E0-E14 (Blum et al., 2017).

lung surfactant-relevant genes are influenced by perinatal microbial exposure. Our data shows that 17 out of our list of 23 lung surfactant-relevant genes are influenced by perinatal microbial exposure: SFTPA1, SFTPB, SFTPC, SFTPD, ABCG1, ABCA1, ABCA3, Bach2, CHKA, PLPP2, Csf2rb, Csf1r-ps, Csf2rb2, Pla2g2a, Pla2r1, Pla2g6, Pla2g12a.

The surfactant proteins SP-A, B, C and D are expressed prenatally before the end of the pseudoglandular stage E16 in mice (Perl et al., 2002) and relatively early in human fetal lung development around week 13 (Liley et al., 1989). Homeostasis and composition of functional pulmonary surfactant is crucial to an operative respiratory surface during the mechanical conditions imposed by breathing, and surfactant properties are linked to the respiratory rate and age (Bernhard et al., 2001; Lopez-Rodriguez and Pérez-Gil, 2014). The hydrophilic proteins, SP-A and SP-D, are involved in lung innate immunity, and the hydrophobic proteins, SP-B and SP-C, are essential for the surface active function of surfactant (Olmeda et al., 2017). We have previously shown some lung microbiota differences between Sftpd+/+ and Sftpd−/− knockout mice in several biologically relevant phylogroups such as *Staphylococcus, Lactobacillus* and *Bifidobacterium* (Barfod et al., 2017).

ATP-binding cassette transporter genes are highly expressed in the lungs compared to many other organs (Langmann et al., 2003). The ABCA1, ABCG1 and ABCA3 are all involved in movement of cholesterol and phospholipids from lung cells, where the ABCA3 is specifically involved in pulmonary surfactant production (Chai et al., 2017). Severe surfactant deficiency in neonates and some forms of Interstitial lung disease (ILD) are the consequence of disturbed lung surfactant homeostasis. This can be due to mutations in the SP-B, SP-C, or ABCA3 genes (Lopez-Rodriguez and Pérez-Gil, 2014; Whitsett et al., 2015). Inactivating the ABCA3 gene causes the mice to die shortly after birth as they are unable to open their lungs (Fitzgerald et al., 2007). It has also been shown that mice lacking ABCG1 have altered lamellar body and surfactant homeostasis and have disturbances in the lung microbiome (Vallim et al., 2015). In our data, the ABCG1 is upregulated in the BI04 animals compared to GF animals, with the largest changes in the male mice.

The granulocyte-macrophage colony stimulating factor genes: Csf2rb, Csf1r-ps, Csf2rb2, are essential for maturation of alveolar macrophages. In addition, they regulate the phospholipid and cholesterol turnover mediated by the ABCG1and ABCA1 lipid transporter genes and are thus critical to surfactant metabolism (Malur et al., 2011). As alveolar macrophages are essential for approximately 20% of surfactant lipids catabolism and clearance (Rider et al., 1992), it would have been interesting to discern the macrophage gene expression from other cell types in our study.

The genes Pla2g2a, Pla2r1, Pla2g6, Pla2g12a belong to a group of more than 30 enzymes that possess PLA2 or related activity found in mammals. A third of these belong to the secreted sPLA2 family (Touqui and Alaoui-El-Azher, 2001) that regulate eicosanoid formation, hydrolyze phospholipids in cell membranes and extracellular structures like surfactant, and have been linked to lung diseases like asthma (Nolin et al., 2016). The surfactant-secreted phospholipase A2 (Pla2g2a) has been linked to respiratory outcome in preterm neonates (De Luca et al., 2020), and Pla2g2a is elevated in the lungs during acute respiratory distress syndrome (ARDS) (Seeds et al., 2012). PLA2R1-induced senescence is related to COPD and lung emphysema pathogenesis, and over-expressing transgenic mice exhibited lung emphysema, fibrosis and pulmonary hypertension (Beaulieu et al., 2021). Pla2g12a is highly expressed in various tissues, but its physiological functions are largely obscure (Murakami et al., 2015).

The lung surfactant-relevant turnover/metabolism pathways are clearly influenced by early-life microbial status and warrant further investigation into microbiome-lung surfactant interaction in early life and during microbiome-altering events later in life e.g. during antibiotics treatment.

We assessed lung surfactant function *ex vivo* for two essential properties, i.e. the ability to adsorb and reduce surface tension at an air liquid interface, and the ability to reduce the minimum surface tension to values low enough to sustain normal breathing at physiologically relevant conditions. Both qualities are essential for the mechanics of breathing, although the reduction in minimum surface tension is dependent upon how much the surface is compressed, thus we kept the compression constant (**Figure 2C**). We found that lung surfactant from GF mice had a lower adsorption surface tension than Cecum-exposed mice, or SPF control mice. This indicates that the lung surfactant from the GF mice more efficiently covers the air-liquid interface in the surfactometer. We also assessed how many compression-expansion cycles it took to reduce the minimum surface tension below 5mN/m. It has been shown previously that inhibition of lung surfactant function delays the ability to reach a low minimum surface tension (Yang et al., 2018) or makes it impossible to reach a low minimum surface tension (Da Silva et al., 2021). The lung surfactant from BI04-treated mice needed to be compressed more times (at the same concentration and compression rate) compared to GF-treated and Cecum-treated mice. This indicates that the lung surfactant from the BI04 mice worked less efficiently. It has not been established how these changes would directly affect breathing patterns *in vivo*.

The breathing patterns recorded *in vivo* by plethysmography reveal that the respiratory rate is inversely correlated with microbial status. GF and BI04 mice have similar low respiratory rates, whereas Cecum and reference SPF mice have similar and higher respiratory rates equivalent to what we have seen in other mouse studies (Sørli et al., 2018). The qualitative and quantitative compositions of lung surfactant are known to vary between species and according to conditions like temperature, breathing pattern, alveolar size, diet developmental stage or hibernation (Lang et al., 2005; Bernhard et al., 2007; Pynn et al., 2010; Suri et al., 2012).

## Limitations and future study

We could have included an SPF group with PNday10 lungs from SPF background (normal) mothers in the lung transcriptomics study, as we did with the plethysmography. Also, due to the observed unexpected sex differences in lung transcripts we should have included higher numbers of animals per group. We did not confirm any transcriptomic results with quantitative methods to access gene expression. The functional pathway analysis revealed a few macrophage-relevant changes and it would have been interesting to isolate the macrophages for a more specific interrogation. Also, a more longitudinal study approach e.g. surfactant transcription during post-natal lung development is warranted.

## Sum-up

Early-life GF mice have similar lung gene transcription compared to gnotophoric or gnotobiotic mice, with only few genes transcribed differently. Among those genes, several are clearly related to surfactant turnover and function. This correlates to physiological changes in respiratory rate and lung surfactant function in littermates at adolescence.

## Conclusion

We show here for the first time that early-life microbial status correlates to altered surfactant gene-transcription and in gnotophoric mice correlates to later-in-life lung surfactant function. We also show respiratory rate changes in GF and gnotophoric mice compared to gnotobiotic and SPF mice. This warrants further investigation into microbiome lung surfactant interactions in disease and health.

## Data availability statement

The datasets presented in this study can be found at: https://doi.org/10.5281/zenodo.6815171.

## Ethics statement

The animal study was reviewed and approved by The experiments were carried out in accordance with the Danish Animal Experimentation Act (LBK nr 474 of 15/05/2014 and BEK nr 12 of 07/01/2016) and the EU-directive 2010/63/EU on the protection of animals used for scientific purposes. The study was approved by the Animal Experimentation Inspectorate, Ministry of Food, Agriculture and Fisheries of Denmark (License No. 2018-15-0201-01531).

## Author contributions

KB conceived and designed the study, obtained funding, performed the mouse work and drafted the paper. JCL contributed with transcriptomics analysis, tools and wrote parts of the paper. SSKH performed RNA extraction and reviewed the paper. LZ was an integral part of the GF mouse work and reviewed the paper. AH, reviewed the paper. JS and SS designed and performed the lung surfactant analysis; collected the surfactant ex vivo data and reviewed the paper. All authors contributed to the article and approved the submitted version.
