# Supplementary figures and images for: The impact of bacterial exposure in early life on lung surfactant gene expression, function and respiratory rate in germ-free mice
Source: Front Microbiomes. 2023 Mar 6;2:1085508. doi: 10.3389/frmbi.2023.1085508 (PMC12993578; doi:10.3389/frmbi.2023.1085508)

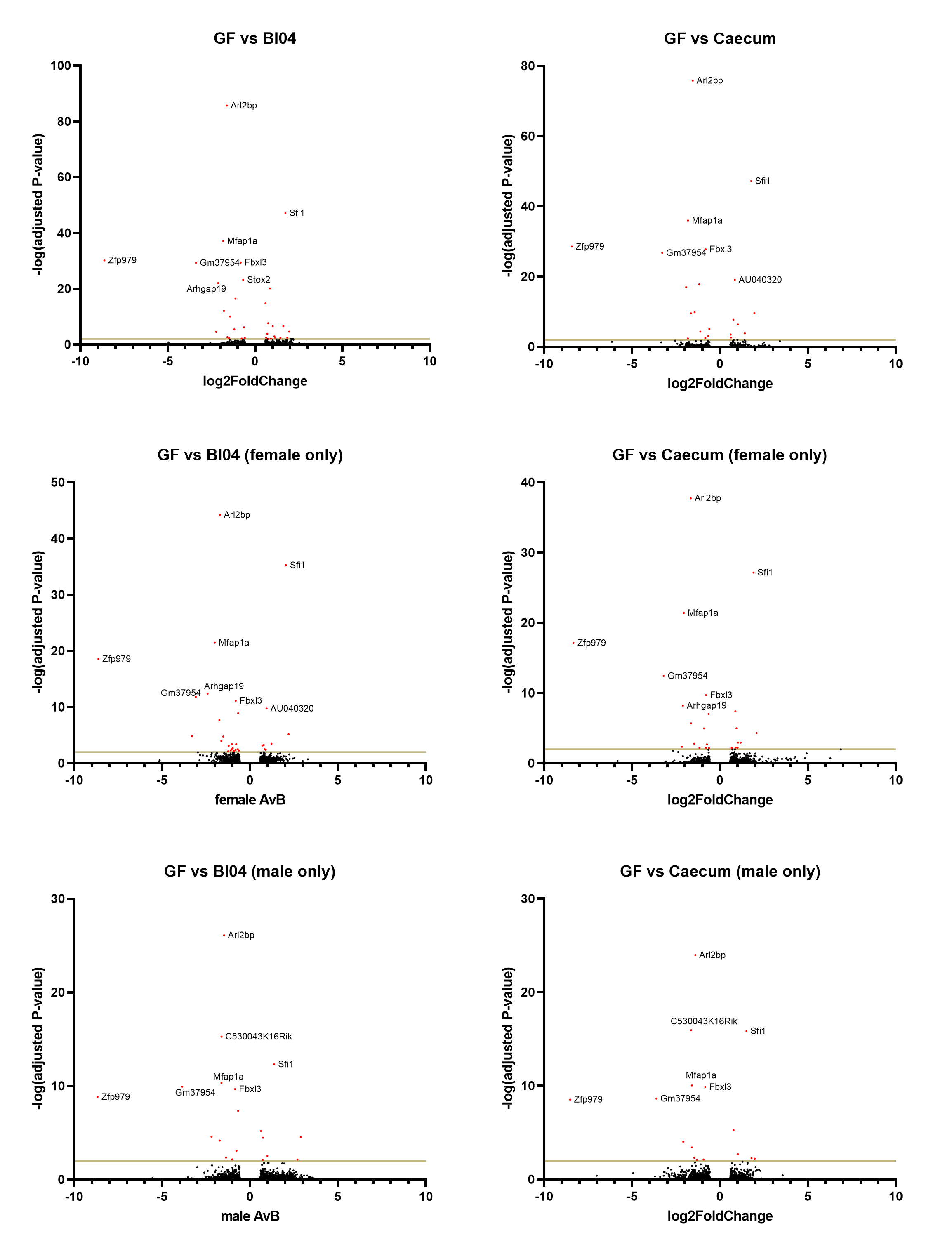

Supplement: Supplementary file 1 [file Image_1.tif]

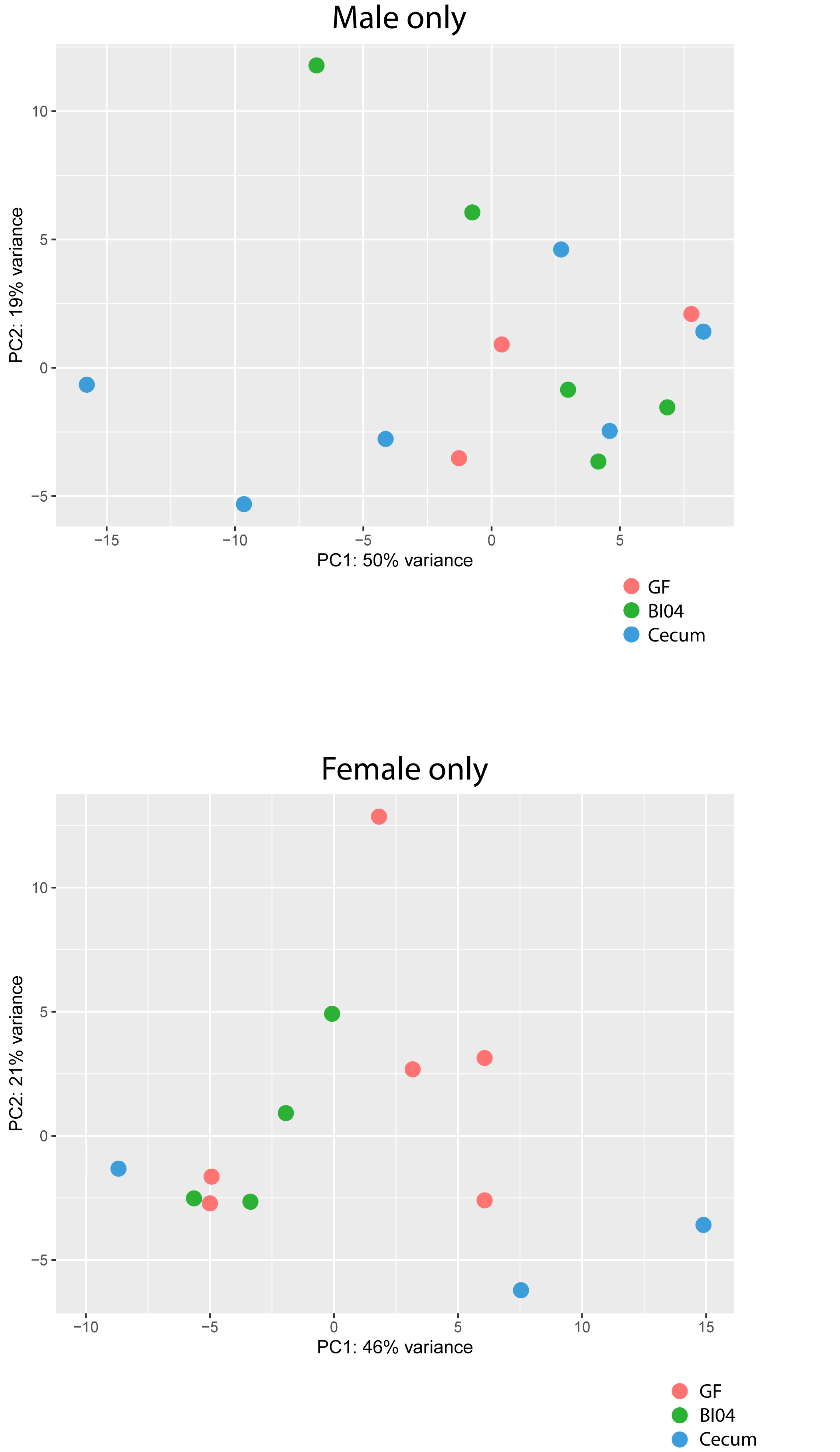

Supplement: Supplementary file 2 [file Image_2.tif]

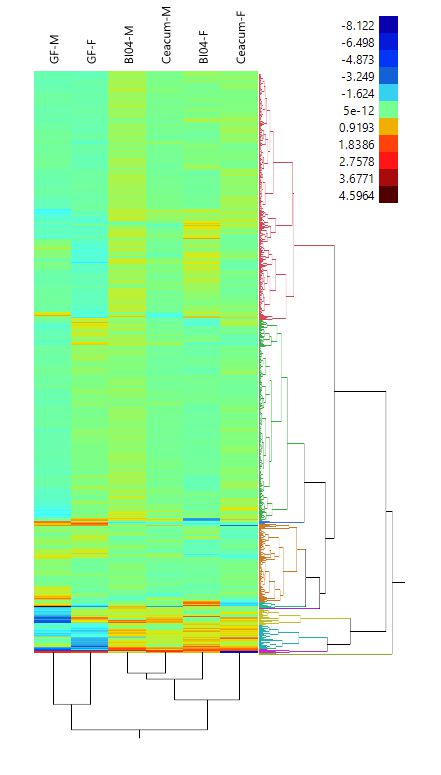

Supplement: Supplementary file 3 [file Image_3.jpeg]
